# The Effects of a Novel Nutraceutical Composition Containing Coenzyme Q_10_, Resveratrol, Extracts of *Ginkgo biloba* Leaf, Garlic Bulb, Red Yeast Rice, and Bovine Colostrum on Cardiac Dysfunction

**DOI:** 10.1002/fsn3.70926

**Published:** 2025-09-12

**Authors:** Sixi Ma, Mingtao Chen, Xuye Lai, Yingying Gu, De Hu, Ying Guo, Lili Yang

**Affiliations:** ^1^ Guangdong Provincial Key Laboratory of Food, Nutrition and Health, Department of Nutrition, School of Public Health Sun Yat‐Sen University Guangzhou People's Republic of China; ^2^ Department of Clinical Research, State Key Laboratory of Oncology in South China, Guangdong Provincial Clinical Research Center for Cancer, Collaborative Innovation Center for Cancer Medicine Sun Yat‐Sen University Cancer Center Guangzhou People's Republic of China

**Keywords:** cardiac dysfunction, cardiovascular disease, hypercholesterolemia, immune function, inflammation

## Abstract

With heart disease being the leading cause of death worldwide, cost‐effective therapies supporting healthy cardiac function are highly needed. Oxidative stress and hypercholesterolemia are risk factors in the pathogenesis of cardiac dysfunction. Many nutraceutical compositions containing Coenzyme Q_10_, resveratrol, extracts of *Ginkgo biloba* leaf, Garlic Bulb, red rice yeast, and bovine colostrum appear to have the potential to improve cardiac function. In the present study, we aimed to evaluate whether a new combination containing the abovementioned nutraceuticals (Cardio) is protective against damage to cardiac function. In this study, C57BL/6J mice were divided into groups of control, high‐fat‐cholesterol diet fed (HFHC), and HFHC diet + Cardio (Cardio group), and fed for 16 weeks. Parameters of plasma lipid levels, inflammation, oxidative damage, and cardiac function were measured. Cardio treatment significantly inhibited hypercholesterolemia, reduced levels of cardiac damage markers including LDH and CK, improved cardiac function, and reduced HFHC diet‐induced increased levels of inflammatory cytokines including TNF‐α and IL‐1β. Our data showed that Cardio has a protective effect on cardiac dysfunction probably by ameliorating hypercholesterolemia, inflammation, and oxidative stress.

## Graphical abstract

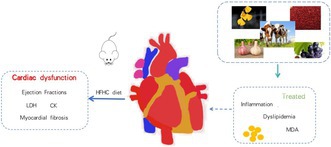

## 1 Introduction

Heart disease is the leading cause of death in the United States. According to the data of Centers for Disease Control and Prevention, one person dies every 33 s in the United States from cardiovascular disease, which costs the United States about $239.9 billion each year from 2018 to 2019. It is well known that the regenerative capacity of cardiomyocytes is weak. When cardiac dysfunction occurs, the normal cardiac structure is difficult to get completely restored (Cheng et al. 2022; Gaggini and Vassalle 2023). Therefore, it is urgent to find an effective scheme to prevent cardiac dysfunction.

There are common risk factors in cardiac dysfunction, such as high levels of oxidative stress (Valenzuela et al. 2023), postoperative chemotherapy (Shah et al. 2023) and abnormal cholesterol or triglyceride levels (Cheung et al. 2024; Giroud et al. 2022). A growing body of evidence for the vital role of chronic low‐grade inflammation in various cardio dysfunctions has recently been demonstrated (Bahrar et al. 2024). Innate immune signals modulate the composition of intestinal flora (Chakaroun et al. 2023), which affects multiple host metabolic processes. Cardiovascular risk increases in the early stage of NAFLD or other metabolic diseases (Kasper et al. 2021), while cardiac dysfunction is largely preventable through nutraceuticals. Extensive research has focused on using nutraceuticals in treating and preventing cardiac dysfunction (Li et al. 2023; Pourbagher‐Shahri et al. 2021; Renke et al. 2023), via mechanisms including lipid‐lowering effect, antioxidant and anti‐inflammation, antiplatelet coagulation, boosting immunization, and vasodilation, etc.

Red yeast is a bioactive ingredient that can effectively lower blood lipids (Cicero et al. 2021; Trogkanis et al. 2024) and is formed by the fermentation of japonica rice by Aspergillus oryzae. Red yeast has been used to reduce lipids for many years (Li et al. 2021). Monacolin K is the active ingredient in red yeast, which exerts a competitive inhibitory effect, reduces endogenous cholesterol formation, and lowers blood lipids (Buzzelli et al. 2024), with a structure similar to the key enzyme in the cholesterol synthesis pathway, HMG‐CoA reductase (Banach et al. 2022; Cicero et al. 2021). Garlic ( *Allium sativum* ) is an aromatic herbaceous plant belonging to the family Amaryllidaceae (Tudu et al. 2022). It has been reported that garlic has the potential to treat hyperlipidemia, hypertension, and cardiovascular diseases (El‐Saadony et al. 2024; Sanie‐Jahromi et al. 2023). In addition, studies found that phenols, organosulfur compounds, and polysaccharides of garlic exerted anti‐inflammatory activity (Melguizo‐Rodriguez et al. 2022).

Bovine colostrum supplementation has been reported as a potential factor in reducing oxidative stress and inflammation (Cieslicka et al. 2022). Higher intakes of folic acid, vitamin B6, and vitamin B12 were also associated with a lower risk of CVD in the general population (Miao et al. 2024). A case–control study demonstrates that high intakes of folate and vitamin B6 are associated with a low risk of CVD among T2D patients (Wu et al. 2023). Therapy with folic acid, vitamin B6, and vitamin B12 lowers the levels of homocysteinemia to prevent atherosclerosis (Prasad 2024). In addition, egg oil prepared from egg yolks shows anti‐inflammatory activity (Xiao et al. 2021). It can be used in the treatment of multiple inflammation‐related diseases such as burns, ulcers, and cheilitis (Shadman‐Manesh et al. 2023).

Coenzyme Q10 (CoQ10), resveratrol, and *Ginkgo biloba* extract are all active ingredients in fighting against oxidative stress (Chen et al. 2024; Qian et al. 2023; Shahcheraghi et al. 2023). Coenzyme Q10, also known as ubiquinone, is an important hydrogen transporter in the oxidative respiratory chain of cells. CoQ10 accepts hydrogen from complex I and complex II, releases protons into the mitochondrial matrix, and transfers electrons to cytochromes (Pu et al. 2023). CoQ10 is involved in oxidative phosphorylation and ATP formation, regulating the cellular redox environment, scavenging free radicals, improving mitochondrial dysfunction, strengthening heart function, regulating blood lipids, and other effects (Watanabe et al. 2023). Resveratrol is a polyphenol extracted from grapes and has good antioxidative effects (Ghavidel et al. 2024). Resveratrol exerts a protective effect in the ejection function of the heart by combining anti‐inflammatory, antioxidant, and anti‐fibrotic actions coupled with improved cardiac stiffness (Zhang et al. 2021). Likewise, *Ginkgo biloba* inhibits oxidative stress and the release of inflammatory cytokines (Qian et al. 2023) and ameliorates obesity‐induced cardiomyopathy by reducing oxidative stress through Nrf2 signaling (Wu et al. 2022).

Despite extensive research on individual nutraceuticals such as CoQ10 and resveratrol, etc., the synergistic cardioprotective effects of the combined application of multiple nutraceuticals remain unexplored. This study is the first to evaluate the preventive potential of a novel nutraceutical composition (Cardio) against diet‐induced cardiac dysfunction. Further, this study proposes a novel, cost‐effective, and multi‐faceted nutritional intervention strategy, offering a potential paradigm in the prevention and management of diet‐induced cardiac dysfunction.

## 2 Materials and Methods

### 2.1 Material and Reagents

The novel nutraceutical composition (Cardio) is a new combination of nutraceuticals, including Coenzyme Q_10_, *Ginkgo biloba* , garlic, Vitamins B12, B6, folate, resveratrol, and red yeast rice extract, cow colostrum extract, and chicken egg yolk extract. The study product is supplied by 4 Life Research LLC (Sandy, UT).

The control diet (No.: TP28602) and the HFHC atherogenic diet [32, 33] (No.: TP28600; 15% fat, 1.25% cholesterol and 0.5% cholate) were from Trophic Animal Feed High‐tech Co. Ltd. (Nantong, China) and stored at 4°C.

### 2.2 Animals

C57BL/6J female mice are one of the most common animal models of cardiovascular disease (Geng et al. 2025; Tadinada et al. 2021; Wang et al. 2022). Five‐week‐old female mice (C57BL/6J) were purchased from Gempharmatech LLC. All mice were housed in an individual pathogen‐free environment under a 12 h light–dark cycle at the Institute for Animal Experimentation, Sun Yat‐Sen University. All experimental procedures were performed in accordance with the guidelines for animal experiments of Sun Yat‐Sen University. After 1 week of adaptive feeding, mice were randomly assigned to three groups based on body weight using a computer‐generated randomization sequence. To be specific, the control group (*n* = 11), which was given control chow plus double‐distilled water by gavage; the model group (HFHC group, *n* = 11), which was given high‐fat chow plus double‐distilled water by gavage; and the intervention group (Cardio group, *n* = 11), which was given high‐fat chow plus a human equivalent dose (152.5 mg/kg/day) of Cardio by gavage. Mice were fed in the SPF environment for 16 weeks.

### 2.3 Echocardiography

After 16 weeks of feeding, transthoracic echocardiography was performed on all experimental mice. The mice were fixed supine on a thermostatic heating plate at 37°C under 1.5% isoflurane inhalation anesthesia. The mouse was shaved on the chest, and the upper abdomen was fully exposed, and the MS‐550D probe of the vevo 3100 small animal ultrasound machine was applied for ultrasound analysis. The left ventricular long‐axis section, and the M mode and B mode data of the mice were recorded. Based on the left ventricular long‐axis section, the probe was rotated 90° clockwise, which was the left ventricular short‐axis section, and the M mode and B mode data were recorded. Ejection fraction (EF), shortening fraction (FS), left ventricular end‐diastolic anterior wall thickness (LVAW; d), left ventricular end‐systolic anterior wall thickness (LVAW; s), left ventricular end‐diastolic posterior wall thickness (LVPW; d), and left ventricular end‐systolic posterior wall thickness (LVPW; s) were analyzed. All the above ultrasound measurements were averaged over 3 consecutive cardiac cycles.

### 2.4 Animal Samples Collection

At the end of the experiment, mice were fasted overnight, anesthetized, blood was withdrawn from the orbits, and the cervical vertebrae were dislocated and executed. Biological samples including heart, liver, intestine, and aorta were collected and preserved in accordance with the experimental plan. Whole blood samples were placed at 26°C for 60 min, centrifuged at 3000 × g for 15 min at 4°C, and centrifuged twice. The supernatant was collected and stored in a −80°C refrigerator for further analysis.

### 2.5 Biochemical Analyses

Serum levels of TC, TG, HDL‐cholesterol (HDL‐C), LDL‐cholesterol (LDL‐C), OX‐LDL, and VLDL; aspartate transaminase (AST) and alanine transaminase (ALT) were measured using commercial assay kits purchased from Jiancheng Bioengineering Institute (Nanjing, China). Cardio risk index analysis: TC/HDL‐C, cardiac risk ratio 1; LDL‐C/HDL‐C, cardiac risk ratio 2; atherogenic index was determined using the Ikewuchi equation [35]. MDA concentration was measured using commercial assay kits from Beyotime biotechnology (China). The levels of TNF‐α, IL‐1β and CRP in the serum were quantified by the enzyme‐linked immunosorbent assay (ELISA) kit (Elabscience Biotechnology Co. Ltd).

### 2.6 Histopathological Analysis

Tissue staining was performed according to a previously reported method [36]. The liver was fixed in 4% paraformaldehyde for hematoxylin and eosin (HE) staining. The heart was fixed in Frozen Embedding Medium for Masson staining and stored at −80°C. The heart tissue was fixed in 4% paraformaldehyde and embedded in paraffin. 5 μm slices were prepared for staining with Masson's trichrome staining. The extent of myocardial fibrosis was observed by Masson's trichrome staining.

### 2.7 Western Blot Analysis

Total proteins were extracted via RIPA containing protease inhibitor PMSF (Beyotime, Shanghai, China) and PhosSTOP phosphatase inhibitor (Beyotime, Shanghai, China). The protein concentration was measured using a BCA assay kit (Beyotime, Shanghai, China). Antibodies against p‐eNOS (S1177) and eNOS were from (Abclonal, China). Protein signals were visualized using an enhanced chemiluminescence detection system according to the manufacturer's instructions (ECL, Thermo Fisher Scientific Waltham, MA, USA).

### 2.8 Statistical Analysis

The collected data were analyzed using GraphPad Prism 9 and expressed as Mean ± Standard Error. Results were evaluated through one‐way analysis of variance (ANOVA) followed by Dunnett's multiple comparisons test. Statistical significance was set at *p* < 0.05.

## 3 Results

### 3.1 Cardio Alleviates the Elevated Levels of Circulating Lipids Induced by HFHC Diet In Vivo

**FIGURE 1:**
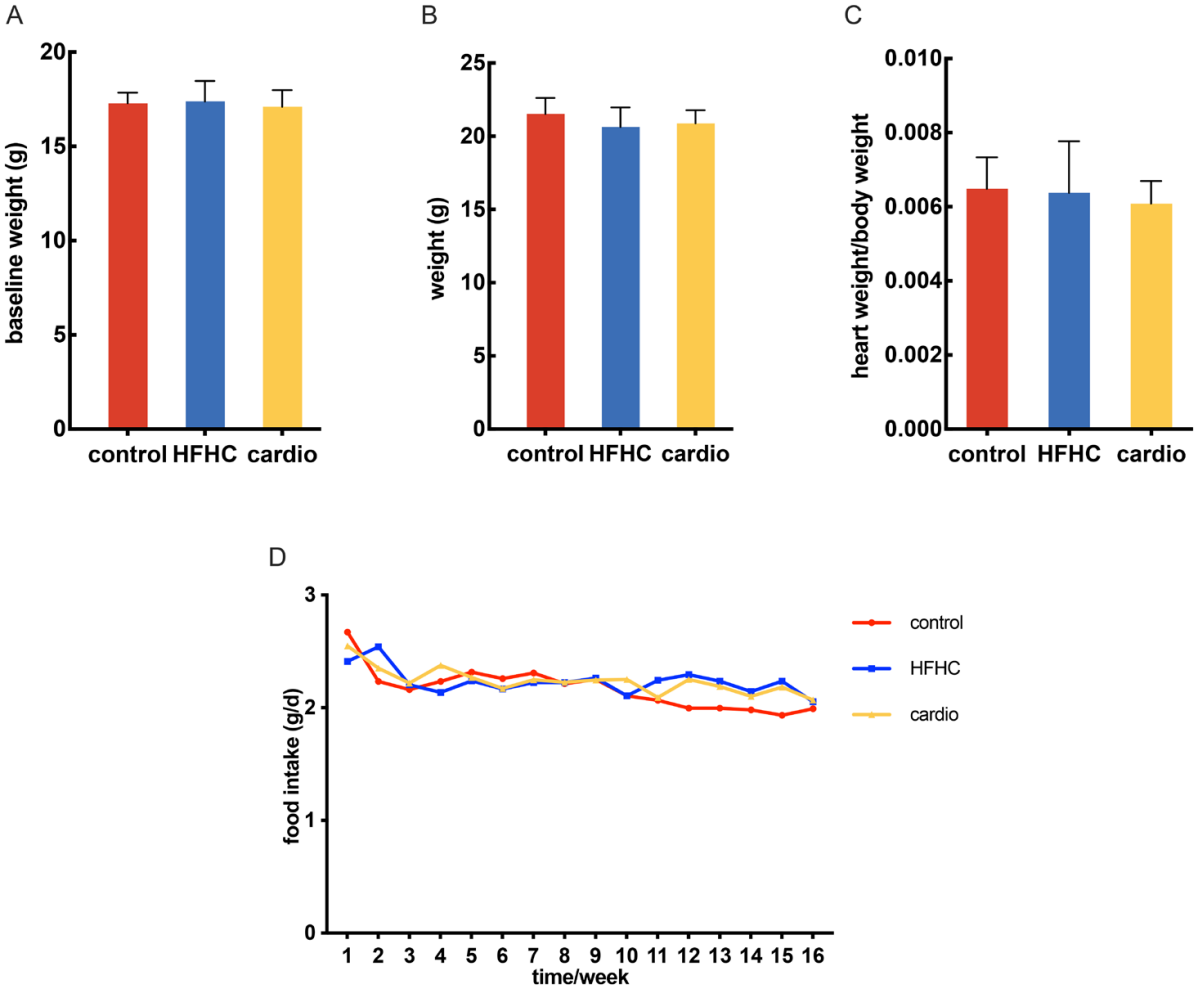
The effects of Cardio supplementation on body weight, food intake, and heart weight. (A) Effects of Cardio on baseline weight of mice compared with the HFHC group. (B) Effects on the weight of mice. (C) Effects on the heart weight of mice. (D) Effects on the food intake of mice. *n* = 11.

**FIGURE 2:**
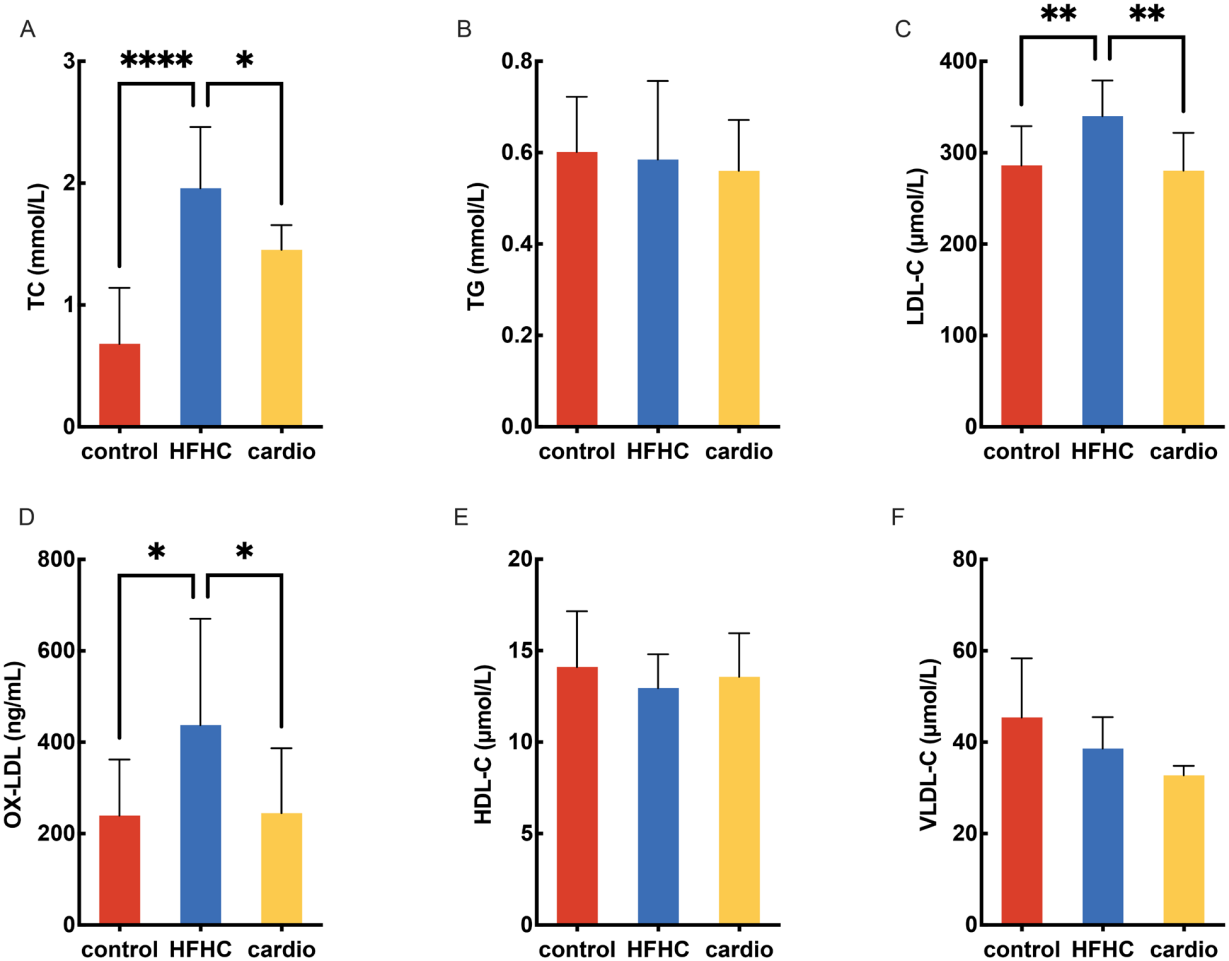
Analysis results of plasma lipid parameters. (A‐F) Levels of TC, TG, LDL‐C, HDL‐C, VLDL‐C, and OX‐LDL. *n* = 11. Compared with the HFHC group, *p < 0.05, **p < 0.01, ***p < 0.001, ****p < 0.0001.

To explore whether Cardio supplementation can protect against HFHC‐induced disorders of lipid metabolism in mice, Cardio (152.5 mg/kg.bw) was applied to HFHC‐fed mice for 16 weeks. The body weight of mice steadily increased among all the groups with no difference (Figure 1A,B). Food intake was similar between the control, Cardio, and HFHC groups (Figure 1C). The blood lipid profile significantly differed between mice in the control group and those fed an HFHC atherogenic diet (Figure 2A–F). The levels of TC, LDL‐C, and OX‐LDL were significantly increased, but those of TG, VLDL‐C, and HDL‐C did not change when comparing HFHC to the control group. The Cardio interventions appeared to alleviate the dyslipidemia caused by the HFHC diet. The calculated cardiac risk ratio 1 was significantly increased in the HFHC group, which was inhibited by the pretreatment with Cardio. Regarding cardiac risk ratio 2, there were no differences among the groups (Table 1). These results suggest that Cardio has protective effects on lipid metabolism and prevents cardiac risk in mice.

**TABLE 1:** Effect of Cardio on plasma parameters in mice.

Parameters	Control	HFHC	Cardio
Cardiac risk ratio 1 (TC/HDL‐C)	0.053 ± 0.0484****	0.154 ± 0.041	0.104 ± 0.024*
Cardiac risk ratio 2 (LDL‐C/HDL‐C)	0.021 ± 0.006	0.026 ± 0.006	0.022 ± 0.004

*Note:* Data are shown as mean ± SE (*n* = 11).

Abbreviations: LDL‐C/HDL‐C, cardiac risk ratio 2; TC/HDL‐C, cardiac risk ratio 1.

* *p* < 0.05.

**** *p* < 0.0001 compared to HFHC group.

### 3.2 Cardio Attenuated HFHC Diet‐Induced Increase of Inflammatory Cytokines

**FIGURE 3:**
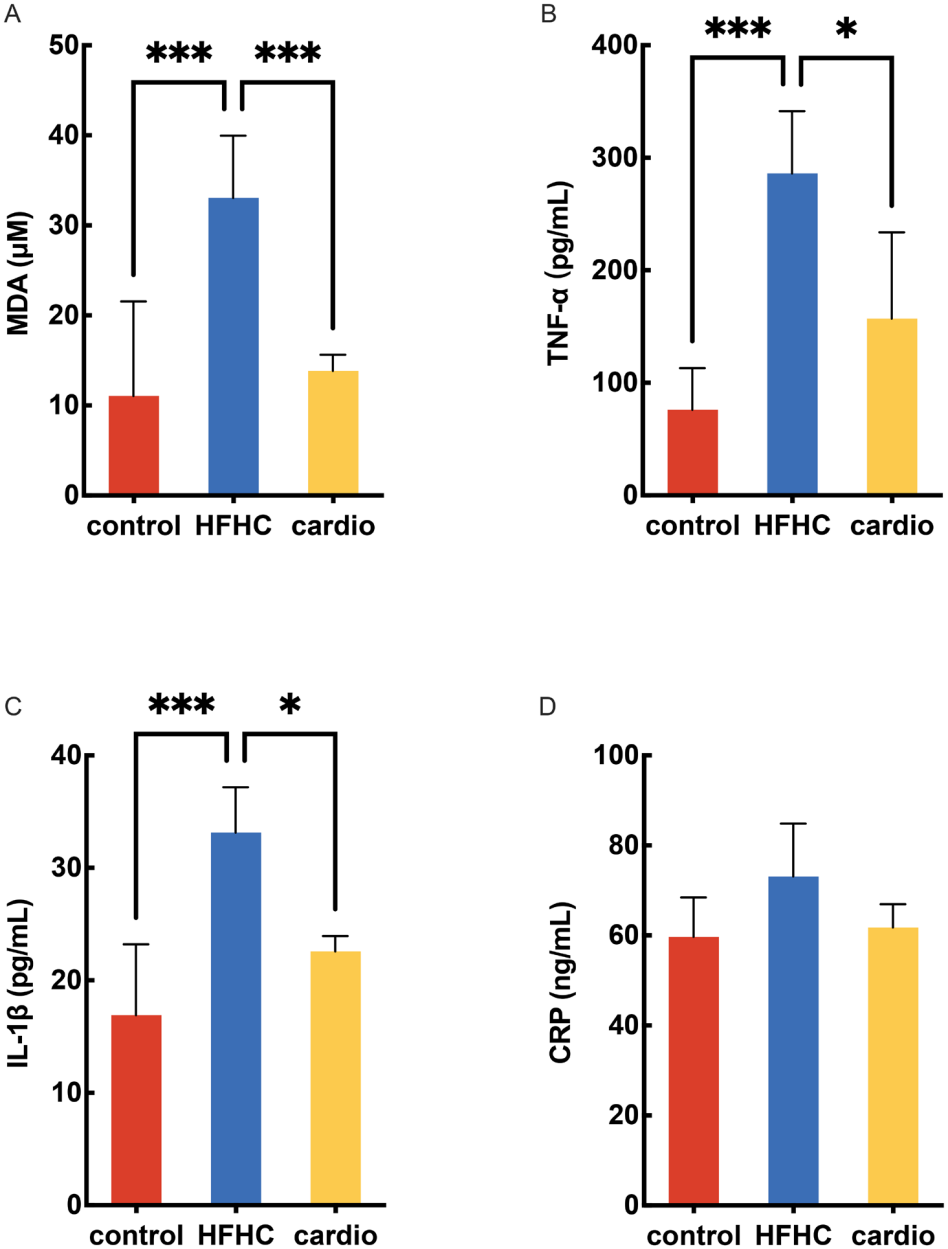
Effects of Cardio on the levels of MDA and pro‐inflammatory cytokines. (A) Malondialdehyde. (B) TNF‐α: Tumor necrosis factor‐α concentration. (C) IL‐1β: Interleukin‐1β concentration. (D) CRP: C‐reactive protein. Compared with the HFHC group, *p < 0.05, ***p < 0.001.

Inflammation has a central role in the development and progression of cardiovascular disease (CVD) (Henein et al. 2022). Oxidative stress is a known trigger for chronic inflammation (Kayesh et al. 2025). Malondialdehyde (MDA) has been widely used for many years as a convenient biomarker for oxidative stress (Mongirdiene et al. 2023). Our results showed that the HFHC diet induced an increasing level of MDA (Figure 3A). When we supplied Cardio, the level of MDA significantly decreased. The levels of pro‐inflammatory cytokines, including tumor necrosis factor‐α (TNF‐α) and interleukin‐1β (IL‐1β) in HFHC diet treatment were significantly higher than those in the control group (Figure 3B,C). Although there was no statistical significance in the levels of serum C‐reactive protein (CRP) among the groups, it was evident that Cardio had a tendency to reduce CRP in mice (Figure 3D).

### 3.3 Cardio Improved Cardiac Function in Mice

**FIGURE 4:**
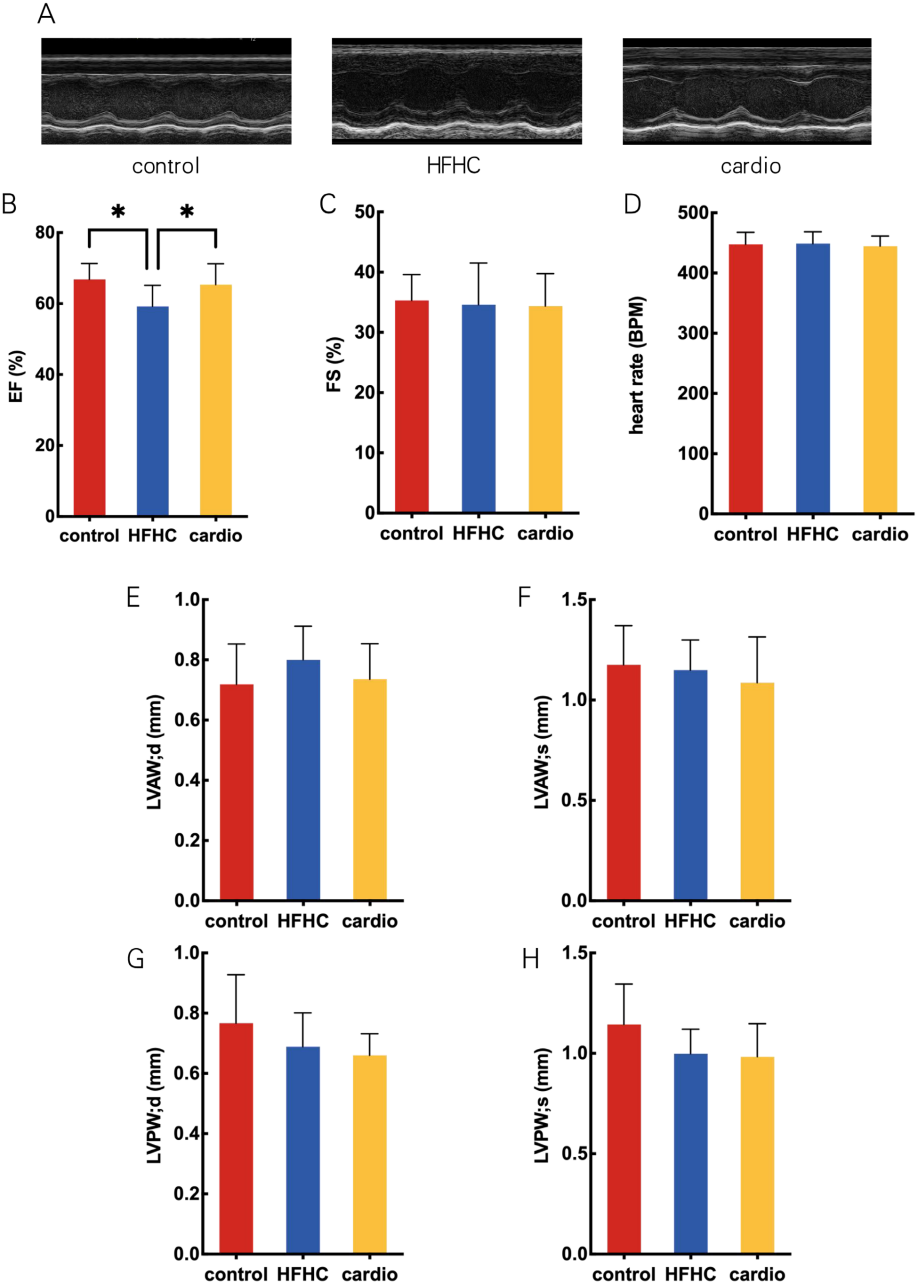
Effect of Cardio on cardiac function in mice. (A) Representative microphotograph of M‐mode ultrasound images. (B) Ejection fraction. (C) Shortening fraction. (D) Heart rate. (E) Left Ventricular End Diastolic Anterior Wall Thickness (LVAW; d). (F) Left Ventricular End Systolic Anterior Wall Thickness (LVAW; s). (G) Left Ventricular End Diastolic Posterior Wall Thickness (LVPW; d). (H) Left Ventricular End Systolic Posterior Wall Thickness (LVPW; s). Compared with HFHC group, *p < 0.05.

There was a significant decrease in ejection fraction (EF) in the HFHC group compared to the control group in mice when ensuring that the heart rate level of each group was around 450 BPM (Figure 4A,B,D). Cardio supplement increased EF. Compared to mice in the control group, shortening fraction (FS) had no difference in mice of the HFHC group (Figure 4C). These results suggest that Cardio can partially improve the contractile function of mice.

Severe cardiac injury is accompanied by structural changes in the heart, such as cardiac hypertrophy and ventricular enlargement. In this study, echocardiography was used to evaluate cardiac structure in mice. Results suggested that Left Ventricular End Diastolic Anterior Wall Thickness (LVAW; d) was similar between the HFHC group and the control group (Figure 4E). The results were in accordance with Left Ventricular End Systolic Anterior Wall Thickness (LVAW; s), Left Ventricular End Diastolic Posterior Wall Thickness (LVPW; d) and Left Ventricular End Systolic Posterior Wall Thickness (LVPW; s) (Figure 4F–H). These results illustrated that either HFHC diet or Cardio did not change heart structure in mice.

### 3.4 Cardio Mitigated HFHC Diet‐Induced Cardiac Muscle Damage in Mice

**FIGURE 5:**
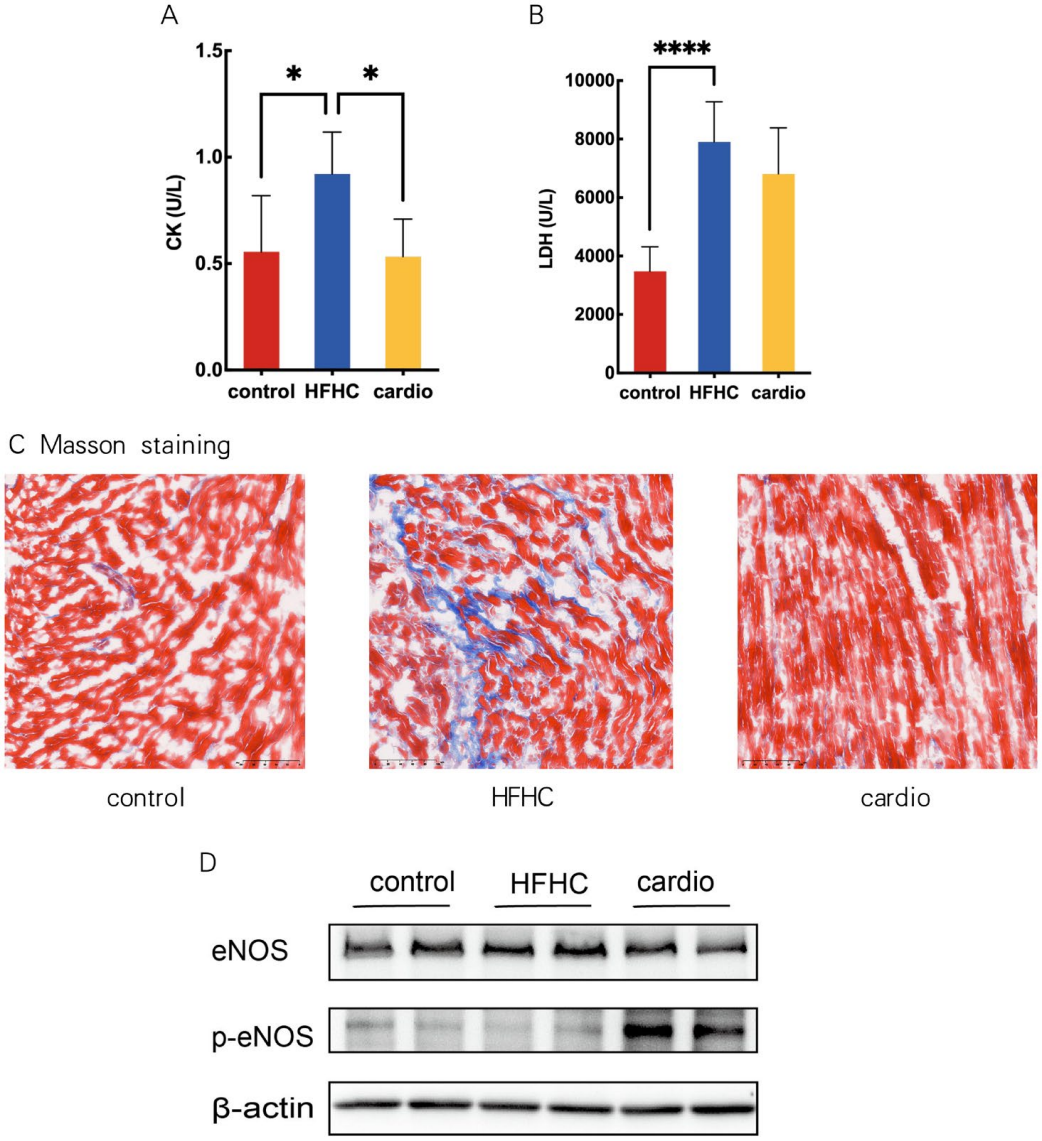
Effect of Cardio on cardiac dysfunction in mice. (A) Creatine kinase. (B) Lactate dehydrogenase. (C) Representative microphotograph of heart muscle Masson staining. (D) Heart muscle western blot. *p < 0.05, ****p < 0.0001.

There was a significant increase in creatine kinase (CK) and lactate dehydrogenase (LDH) in the HFHC group compared with the control group. Cardio supplement reduced the level of creatine kinase (Figure 5A,B). The HFHC diet induced myocardial fibrosis, and Cardio ameliorated myocardial fibrosis shown by Masson staining induced by the HFHC diet in mice (Figure 5C). Furthermore, we explored whether the elevated cardiac function by Cardio is related to eNOS activation. The expression of p‐eNOS (S1177) was decreased in the HFHC group compared to control, indicating the decreased activation of eNOS by the HFHC diet, which was elevated in the Cardio group (Figure 5D), suggesting a possible regulatory role of eNOS activation on cardiac function.

### 3.5 Cardio Ameliorates Liver Damage Induced by HFHC Diet

**FIGURE 6:**
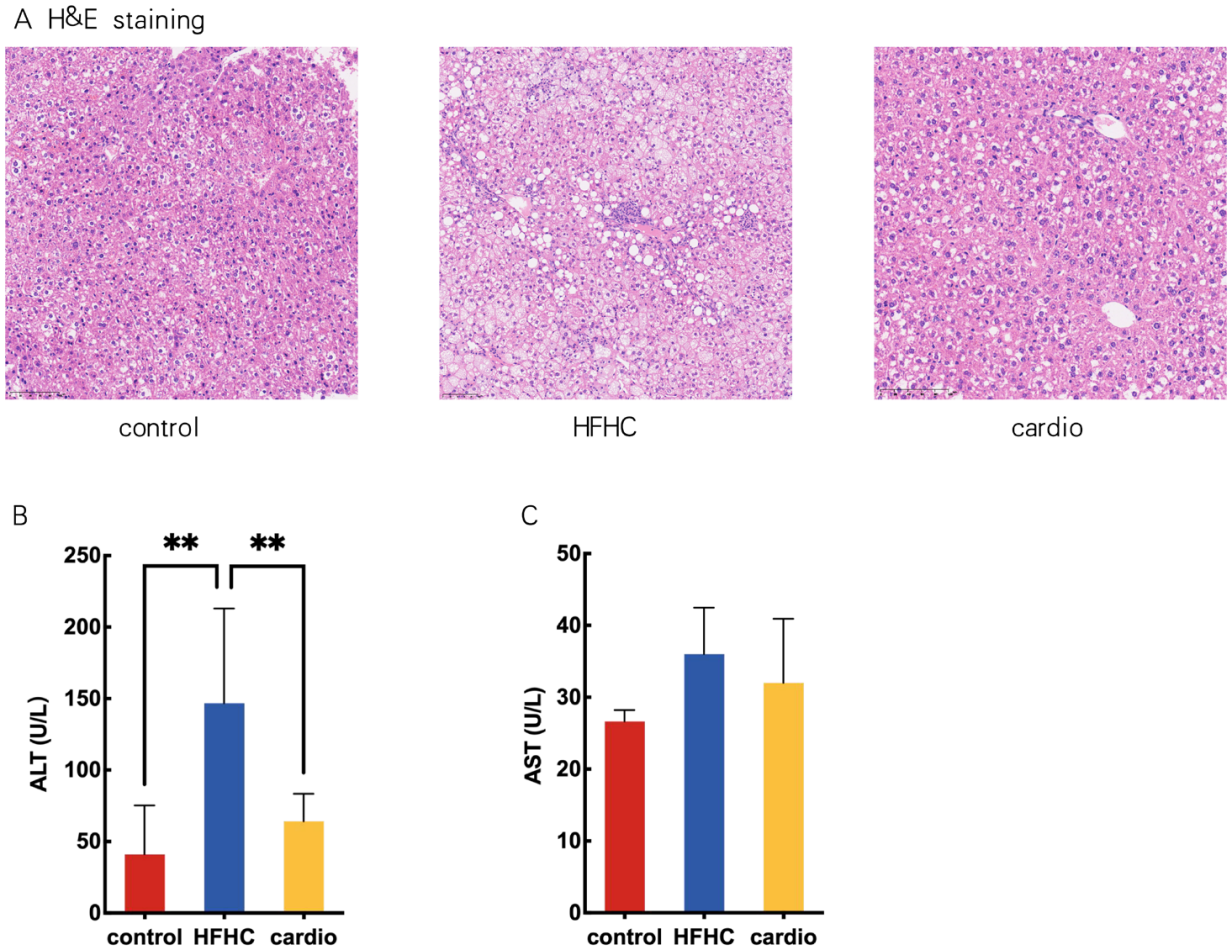
Effects of Cardio on hepatic histopathology and function in mice. (A) Representative microphotograph of liver hematoxylin and eosin (H&E) staining. (B) ALT: Alanine aminotransferase. (C) Level of serum AST: Aspartate aminotransferase. Compared with HFHC group, *p* < 0.01.

Accumulations of lipid and inflammatory cells were increased in the HFHC group compared with the control in H&E staining of liver slices, which were prevented by Cardio supplementation (Figure 6A). The level of ALT in the HFHC group was higher than that of the control. Cardio supplement ameliorated the increase in ALT induced by the HFHC diet (Figure 6B). There was no statistical significance in the level of AST among the three groups (Figure 6C).

## 4 Discussion

Our study found that Cardio exhibits cardioprotective effects in C57BL/6J mice probably by ameliorating dyslipidemia, oxidative stress, and inflammation induced by an HFHC diet.

Individuals with heart disease exhibit significantly elevated dyslipidemia‐related biomarkers, including total cholesterol (TC), low‐density lipoprotein cholesterol (LDL‐C), and cardiac risk ratios (TC/HDL‐C and LDL‐C/HDL‐C) (Princip et al. 2023; Yuan et al. 2024). Therefore, comprehensive lipid profiles or cardiac risk ratios could serve as effective biomarkers for predicting the development and progression of cardiac dysfunction (Princip et al. 2023; Xu et al. 2024). Red yeast rice exerts potent serum cholesterol‐lowering effects, which have been confirmed by several studies (Fukami et al. 2021; Liu et al. 2024). Monacolin K, a bioactive compound in red yeast rice, acts as a competitive inhibitor of 3‐hydroxy‐3‐methylglutaryl‐coenzyme A (HMG‐CoA) reductase, which is a rate‐limiting enzyme in hepatic cholesterol biosynthesis. By suppressing this key enzymatic activity, monacolin K effectively reduces endogenous cholesterol production and plays a critical role in maintaining systemic cholesterol homeostasis (Buzzelli et al. 2024; Fukami et al. 2021). Our findings demonstrate that the Cardio significantly modulates lipid metabolism and lowers the cardiac risk ratio, thereby mitigating the risk of cardiac dysfunction. The effect of reducing the lipid profile of Cardio might be partially due to the lipid‐lowering components, including red yeast rice extract.

Inflammation is known to play an important role in the pathogenesis of cardiac dysfunction (Giroud et al. 2022). The pro‐inflammatory cytokines have a direct effect on metabolic processes (Toldo and Abbate 2024). For example, TNF‐α can ultimately lead to macrophage infiltration and heart muscle damage (Gaggini et al. 2022). Previous research showed that CoQ10 can alleviate inflammation partially via inhibiting the NLRP3/IL‐1β pathway to promote the recovery of cardiac function after myocardial infarction (Pan et al. 2024). Resveratrol is effective in decreasing the levels of inflammatory factors such as TNF‐α and IL‐1β in rats (Ghavidel et al. 2024). Resveratrol has been shown to mitigate aging‐induced cardiac dysfunction by suppressing oxidative stress and inflammation through the Notch/NF‐κB pathway in heart tissue (Wang et al. 2024). *Ginkgo biloba* extract exerted cardioprotective effects by attenuating cardiac inflammation, primarily through suppression of the canonical NF‐κB signaling pathway (Zhang et al. 2022). In our study, we confirmed that the levels of inflammatory cytokines were significantly increased in HFHC diet‐fed mice. While Cardio treatment showed a decrease in the levels of inflammatory cytokines and improved the pathological tissue structure, these effects may be attributed to the synergistic modulation of multiple inflammatory pathways by its bioactive components. This result supplies another piece of evidence, as published in those papers, suggesting that anti‐inflammatory nutraceuticals have the potential to protect cardiac function (Cao et al. 2021; Fladerer and Grollitsch 2023; Mohammadi et al. 2024).

The significant reduction in serum levels of cardiac injury biomarkers, including CK and LDH, provides direct evidence of Cardio's protective effects on cardiomyocyte integrity. The primary manifestations of cardiac dysfunction included impaired cardiac function, elevated serum levels of CK and LDH, and myocardial fibrosis (Chen et al. 2023; Kim et al. 2021). Our results suggest that Cardio could alleviate hyperlipidemia induced by the HFHC diet. Compared to the HFHC group, the Cardio group increased the ejection fraction, lowered serum levels of CK and LDH, and decreased the degree of myocardial fibrosis induced by the HFHC diet. The mechanism of protecting cardiac health by Cardio may be related to its combination of multiple active ingredients, including Coenzyme Q10, *Ginkgo biloba* , garlic, Vitamins B12, B6, folate, resveratrol, and red rice yeast extract. Elevated CK and LDH are hallmarks of myocardial membrane disruption and energy metabolism dysfunction, often resulting from oxidative stress‐induced lipid peroxidation or inflammatory cytokine‐mediated cell death (Antar et al. 2024). The downregulation of these markers likely reflects preserved mitochondrial function and attenuated myocardium damage by Cardio supplementation, consistent with the observed decrease in MDA and pro‐inflammatory cytokines.

The mechanism by which Cardio improves cardiac function may be related to its promotion of endothelial NO synthase (eNOS) activation in the heart. eNOS activation contributes to the production of Nitric oxide (NO) in endothelial cells and cardiomyocytes (Yang et al. 2023), which exerts pleiotropic cardioprotective effects, including vasodilation, inhibition of platelet aggregation, and suppression of inflammatory responses (Wu et al. 2021). Serine 1177 (S1177) is a phosphorylation activation site for eNOS and can reflect the degree of eNOS activation, while it is also a critical regulator of NO biosynthesis (Jin et al. 2022; Liang et al. 2021). Our findings align with the observed elevation in EF and attenuation of myocardial fibrosis, suggesting Cardio supplementation enhanced myocardial compliance and contractility. These results suggest that the improved cardiac function of Cardio in mice may be related to promoting myocardial eNOS activation. Earlier research has shown that the concurrent use of simvastatin‐loaded nanoparticles and CoQ10‐loaded nanoparticles enhances the Akt‐eNOS signaling pathway and optimizes the balance of NO and reactive oxygen species (ROS) (Saman et al. 2022). *Ginkgo biloba* extract enhances SOD1 expression, thereby regulating the PTEN/PI3K/Akt/eNOS signaling pathway and promoting NO‐mediated endothelial function (Taguchi et al. 2023). Similarly, resveratrol could enhance endothelial function by increasing eNOS (Damay and Ivan 2024). Deodorized garlic increases the H_2_S, promotes the activation of eNOS, and then through the cross‐talk between the H_2_S and the NO systems to protect cardiac function (Perez‐Torres et al. 2022). As a consequence, the multi‐component nature of Cardio likely drives synergistic activation of eNOS through complementary pathways mentioned above.

In our study, we used female C57BL/6J mice to study the effect of HFHC diet and Cardio supplementation. It was known that both C57BL/6J mice and ApoE−/− mice are representative animal models of dyslipidemia and cardiac dysfunction (Chaix et al. 2024; Cho et al. 2024; Christodoulou et al. 2024; Maurya et al. 2023); we chose to feed C57BL/6J mice with a high fat high cholesterol diet to simulate the natural progression of dyslipidemia and cardiac dysfunction in human beings. HFHC diet is one of the most common diets to induce dyslipidemia and cardiac dysfunction (Jose et al. 2025; Mei et al. 2022; Shiraishi et al. 2024). As far as the gender of mice is concerned, it was reported that female mice are more susceptible to HFD‐induced cardiac dysfunction (Joseph et al. 2022). These facilitate the application of female C57BL/6J mice in the investigation of the protective effects of Cardio on cardiac function.

In this study, we did not see a higher body weight in the HFHC group at week 16, which is due to using an isoenergetic control diet in comparison to the HFHC diet. Since energy intake was similar in all three groups, mice in all three groups have similar body weight at the end of the experiment.

In conclusion, this study demonstrated that Cardio, as a multi‐component nutraceutical, effectively mitigates diet‐induced cardiac dysfunction through integrated modulation of lipid metabolism, oxidative stress, and inflammation. Its multi‐target cardioprotective function positions it as a promising candidate for preventive strategies in populations at high risk of cardiovascular disease, particularly those with metabolic syndrome or hypercholesterolemia. Future clinical trials are warranted to validate these preclinical observations and optimize dosing regimens for translational applications.

## Author Contributions

**Sixi Ma:** methodology (equal), visualization (equal), writing – original draft (equal). **Mingtao Chen:** software (equal), visualization (equal), writing – review and editing (equal). **Xuye Lai:** investigation (equal), validation (equal). **Yingying Gu:** formal analysis (equal), investigation (equal). **De Hu:** data curation (equal), resources (equal). **Ying Guo:** project administration (equal), supervision (equal). **Lili Yang:** conceptualization (equal), funding acquisition (equal), writing – review and editing (equal).

## Ethics Statement

The study was conducted in accordance with the Ethics Committee of Sun Yat‐sen University. All animal experiments in this study were approved by the Animal Care and Protection Committee of Sun Yat‐sen University.

## Conflicts of Interest

The authors declare no conflicts of interest.

## Data Availability

The data presented in this study are available on request from the corresponding author.
